# Reply to “Revisiting Cryoablation for AVNRT: A Commentary on Safety, Recurrence, and Clinical Implications”

**DOI:** 10.1002/joa3.70214

**Published:** 2025-10-24

**Authors:** Shinichi Tachibana, Yasuteru Yamauchi

**Affiliations:** ^1^ Department of Cardiology Japan Red Cross Yokohama City Bay Hospital Yokohama Kanagawa Japan

**Keywords:** atrioventricular block, atrioventricular nodal re‐entrant tachycardia, catheter ablation, cryoablation, slow pathway

Dear Editor,

We are grateful to Ahmed Z et al. for their interest in our previous article [1] and for pointing out significant limitations. Our study compared the dynamics of atrioventricular block (AVB) during slow pathway modification for atrioventricular nodal reentrant tachycardia (AVNRT) using cryoablation versus radiofrequency ablation (RFA) and aimed to describe how AV conduction disturbance evolves intra‐procedurally and how this relates to procedural safety near the His‐bundle potential.

We acknowledge that transient AVB occurred more frequently with cryoablation than with RFA (CRYO group: 24.1% vs. RFA group: 6.4%, *p* < 0.01). However, no cases of permanent AVB were observed in the CRYO group, whereas three patients in the RFA group required pacemaker implantation. In our interpretation, safety refers to the absence of permanent AVB and the fully reversible nature of the AV conduction disturbances. Therefore, we argue that although operator caution is warranted due to the high incidence of transient AVB, the reversibility of AV conduction disturbances supports the safety of cryoablation procedures near the His‐bundle potential.

Ahmed et al. question whether the longer time to second‐ or third‐degree AVB with cryoablation (6.6 ± 3.7 s vs. 1.2 ± 0.3 s with RFA) translates into actionable safety benefits. In our protocol, continuous PR‐interval monitoring and high‐rate atrial burst pacing with 1:1 fast‐pathway conduction were systematically used to promptly detect fast‐pathway disturbances; freezing was stopped immediately if the PR‐interval prolonged by > 50 ms or AVB occurred during cryoablation, and the catheter was withdrawn. Thus, the longer delay to AVB with cryoablation (6.6 ± 3.7 s vs. 1.2 ± 0.3 s with RFA) provided operators with a real‐time safety margin, allowing them to interrupt freezing before irreversible injury developed and prevent progression to permanent AVB, which may explain why no permanent AVB occurred in the CRYO group.

We acknowledge the higher overall AVNRT recurrence in the CRYO group compared with the RFA during a median 221 ± 186‐day follow‐up (9.5% vs. 3.4%, *p* < 0.01). Two contextual factors may account for this finding. First, successful ablation sites located above the coronary sinus roof were more common with cryoablation (67.9% vs. 34.3%, *p* < 0.01), reflecting a safety‐driven preference for more superior targeting near the His‐bundle potential. Second, outcomes varied by anatomical region; for example, in region III (Figure 1 of our article), AVNRT recurrence was significantly more frequent in the CRYO group (12.2% vs. 3.4%, *p* < 0.001). These findings suggest that anatomy and target selection critically influence outcomes and should guide individualized procedural strategies. As we mentioned in our limitations, we did not capture detailed patient anatomy or cooling dynamics, which may relate to lesion durability. These aspects warrant investigation in future prospective studies.

**FIGURE 1:**
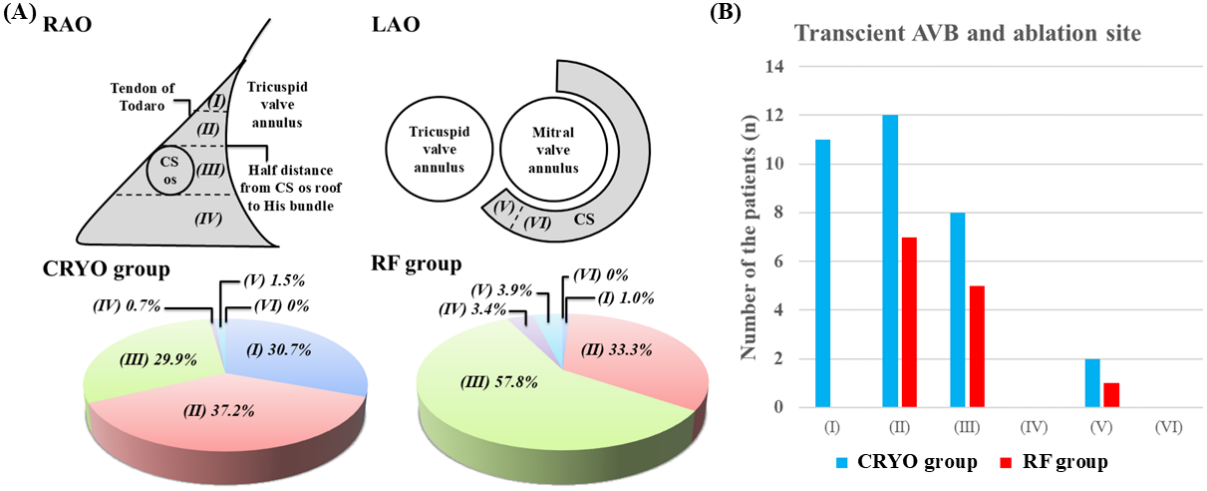
(A) The anatomical location of the successful site was categorized into six regions: (i) The upper part of Koch's triangle, between the coronary sinus (CS) ostial roof and the His bundle, (ii) The lower part of Koch's triangle, between the CS ostial roof and the His bundle, (iii) The region at the same height as the CS in Koch's triangle, (iv) The region below the bottom of the CS, (v) inside the CS ostium, and (v) inside the CS. (B) The anatomical location of the ablation site and its relationship to transient AVB. Reproduced from Tachibana et al. [1].

Moreover, a longer follow‐up period will be required to fully characterize long‐term AV nodal safety. Importantly, no patient with intra‐procedural transient AVB developed permanent AVB after discharge in our cohort, which we believe is a clinically relevant observation despite the brief follow‐up period.

In summary, our data support cryoablation as a reasonable option when ablation near the His bundle is anticipated or in patients with smaller Koch's triangles. The gradual and reversible AV conduction changes observed with cryoablation provide a practical safety margin that allows operators to intervene before permanent AVB develops. At the same time, we recognize that the risk of AVNRT recurrence may be higher depending on anatomical features and ablation technique in the context of cryoablation. Therefore, the choice of energy modality should be individualized, balancing the risk of complications, anatomical constraints, and long‐term efficacy.

## Ethics Statement

This study was approved by the local ethics committees.

## Consent

Written informed consent was obtained from all patients.

## Conflicts of Interest

The authors declare no conflicts of interest.

## Data Availability

The data underlying this article will be shared on reasonable request to the corresponding author.

## References

[1] S. Tachibana , T. Asakawa , Y. Sagawa , et al., “Characteristics of Atrioventricular Block During Slow Pathway Ablation for Atrioventricular Nodal Re‐Entrant Tachycardia: A Comparative Study of Cryoablation and Radiofrequency Ablation,” Journal of Arrhythmia 41 (2025): e70072. 40313583 10.1002/joa3.70072 PMC12041887

